# Blood biomarker discovery for autism spectrum disorder: A proteomic analysis

**DOI:** 10.1371/journal.pone.0302951

**Published:** 2024-12-19

**Authors:** Laura Hewitson, Jeremy A. Mathews, Morgan Devlin, Claire Schutte, Jeon Lee, Dwight C. German

**Affiliations:** 1 The Johnson Center for Child Health and Development, Austin, TX, United States of America; 2 Bioinformatics & Computational Biology Program, Departments of Mathematical Sciences and Biological Sciences, University of Texas at Dallas, Dallas, TX, United States of America; 3 Lyda Hill Department of Bioinformatics, UT Southwestern Medical Center, Dallas, TX, United States of America; 4 Department of Psychiatry, UT Southwestern Medical Center, Dallas, TX, United States of America; Fondazione Policlinico Universitario Gemelli IRCCS, ITALY

## Abstract

Autism spectrum disorder (ASD) is a neurodevelopmental disorder characterized by deficits in social communication and social interaction and restricted, repetitive patterns of behavior, interests, or activities. Given the lack of specific pharmacological therapy for ASD and the clinical heterogeneity of the disorder, current biomarker research efforts are geared mainly toward identifying markers for determining ASD risk or for assisting with a diagnosis. A wide range of putative biological markers for ASD are currently being investigated. Proteomic analyses indicate that the levels of many proteins in plasma/serum are altered in ASD, suggesting that a panel of proteins may provide a blood biomarker for ASD. Serum samples from 76 boys with ASD and 78 typically developing (TD) boys, 2–10 years of age, were analyzed to identify possible early biological markers for ASD. Proteomic analysis of serum was performed using SomaLogic’s SOMAScan^TM^ assay 1.3K platform. A total of 1,125 proteins were analyzed. There were 86 downregulated proteins and 52 upregulated proteins in ASD (FDR < 0.05). Combining three different algorithms, we found a panel of 12 proteins that identified ASD with an area under the curve (AUC) = 0.8790±0.0572, with specificity and sensitivity of 0.8530±0.1076 and 0.8324±0.1137, respectively. All 12 proteins were significantly different in ASD compared with TD boys, and 4 were significantly correlated with ASD severity as measured by ADOS total scores. Using machine learning methods, a panel of serum proteins was identified that may be useful as a blood biomarker for ASD in boys. Further verification of the protein biomarker panel with independent test sets is warranted.

## Introduction

Autism spectrum disorder (ASD), a heterogeneous neurodevelopmental disorder, is characterized by deficits in social communication and social interaction, with restricted, repetitive patterns of behavior, interests, or activities [1]. ASD impacts at least 1 out of every 59 children in the U.S. [2], although this is likely underestimated [3]. Consequently, ASD is associated with considerable personal, family, and societal costs. For these reasons, efforts directed toward determining the underlying pathobiology of ASD, as well as ASD prevention, early diagnosis, and effective treatments, are public health priorities [4].

ASD is currently diagnosed based on behavioral criteria because its underlying disease mechanisms and associated medical, neurological, and psychiatric comorbidities are poorly understood [5–7]. However, the diagnostic methods and screening tools utilized for ASD are somewhat subjective and are difficult to assess in younger children. Early diagnosis is critical because not only are intensive behavioral therapy programs effective in decreasing maladaptive behaviors in many children with ASD [8], the benefits of early intervention are typically greater the earlier the intervention begins [9]. A biological marker that could predict ASD risk, assist in early diagnosis, or even identify potential therapeutic targets has great clinical utility [10–12].

Based on our current understanding of the etiology of ASD, many blood-based biomarker candidates have been investigated [13], including neurotransmitters [14], cytokines [15], markers of mitochondrial dysfunction [12, 16], and markers of oxidative stress and impaired methylation [17, 18]. We have previously demonstrated that thyroid-stimulating hormone (TSH) and interleukin-8 (IL-8) were effective for separating boys with ASD from healthy control subjects, and levels were correlated with the severity of ASD [10]. However, given that idiopathic ASD is a highly prevalent and heterogeneous disorder, and unidimensional ASD biomarker studies have repeatedly met with challenges in reproducibility [11, 19, 20], there is an obvious need to incorporate machine learning in these analyses to more powerfully examine disease status and symptom severity [6, 21]. The use of machine learning in ASD datasets may also allow for more precise, individualized medical care by identifying risk, confirming diagnosis, or guiding responses to treatments [18, 22–25].

The present study was previously published in February 2021 [26]. It was retracted in March 2024 [27] due to an error in the correlation analysis. The correlation analysis was conducted to examine the relationship between protein counts and ASD behavior using Autism Diagnostic Observation Schedule (ADOS) total scores. Since the ADOS was only administered to boys in the ASD group, we randomly assigned ADOS total scores of 1, 2, or 3 to TD boys for the correlation analysis. However, since the ADOS is not a norm-referenced test [28], including the randomly-assigned scores from the TD group was incorrect. The revised correlation analysis presented here therefore includes the calculated ADOS total scores from the ASD group only. This reanalysis resulted in an updated biomarker panel of 12 proteins versus the 9 that were previously published [26] and includes 6 core proteins and 6 additive proteins. The new panel of 12 proteins identified ASD with a slightly more robust AUC than the original publication. The top-20 biological processes from the pathway enrichment analysis have been updated using the 12 optimal proteins in the biomarker panel. While some of the GO Terms have changed, the proteins have pathway significance related to a number of processes associated with immune function in ASD, as described in the original paper. Finally, participants reporting allergies had significantly lower IgD protein counts to those reporting no allergy, and ethnicity was significantly associated with two non-core proteins. Other than the correlation anslysis, no changes were made to the methodology, and the overall conclusion of this paper remain the same as the previous publication [26].

The objective of the present study was to conduct a proteomic analysis of serum from boys with and without ASD using the SomaLogic SOMAScan^TM^ platform, incorporating machine learning of the associated demographic and clinical data, for biomarker discovery.

## Materials and methods

### Participants

The study protocol and subsequent amendments were submitted by The Johnson Center for Child Health and Development (Austin, TX) and approved either by the Austin Multi-Institutional Review Board (for samples collected before October 2016) or IntegReview Institutional Review Board (for samples collected from October 2016 onwards). The study was carried out in accordance with the relevant guidelines and regulations. The recruitment period for this study was 11/22/2010-10/12/2017. Written informed consent was obtained in person from all participants and/or their legal guardians before study participation. Subjects with a genetic, metabolic, or other concurrent physical, mental, or neurological disorder were excluded.

A total of 154 male pediatric subjects were enrolled. The ASD group was comprised of 76 subjects with a mean age of 5.6 years (SD 1.7 years). The TD group was comprised of 78 subjects with a mean age of 5.7 years (SD 2.0 years). The ethnic breakdown was as follows: 73 White/Caucasian, 32 Hispanic/Latino, 17 African American/Black, 5 Asian or Pacific Islander, 23 multiple ethnicities or other, and 4 not reported (Table 1). Co-morbid/clinical conditions and the use of psychiatric medications are summarized in Table 1.

**Table 1:** Demographic data, co-morbid conditions, and use of psychiatric medications in ASD and TD subjects.

	ASD (n = 76)	TD (n = 78)
Age: mean (SD) years	5.6 (1.7)	5.7 (2.0)
Ethnicity		
*White/Caucasian*	33 (45.2%)	40 (51.9%)
*Hispanic/Latino*	26 (35.6%)	6 (7.8%)
*African American/Black*	3 (4.1%)	14 (18.2%)
*Asian or Pacific Islander*	2 (2.6%)	3 (3.9%)
*Multiple ethnicities or Other*	9 (12.3%)	14 (18.2%)
*Not reported*	3 (4.1%)	1 (1.2%)
Co-morbid conditions*		
*None*	38 (52.8%)	58 (75.3%)
*ADHD*	2 (2.8%)	1 (1.3%)
*Allergies*	27 (37.5%)	17 (22.1%)
*Asthma*	2 (2.8%)	0 (0%)
*Celiac Disease*	1 (1.4%)	0 (0%)
*GERD*	1 (1.4%)	0 (0%)
*PTSD*	0 (0%)	1 (1.3%)
*Sleep Apnea*	2 (2.8%)	0 (0%)
*Not reported*	4 (5.6%)	1 (1.3%)
Psychiatric medications^**^**^		
*None*	69 (92%)	75 (97.4%)
*Anti-depressant*	4 (5.3%)	0 (0%)
*Anti-psychotic*	1 (1.3%)	1 (1.3%)
*Sedative*	1 (1.3%)	0 (0%)
*Stimulant*	1 (1.3%)	1 (1.3%)
*Not reported*	1 (1.3%)	1 (1.3%)

*Some participants reported multiple co-morbid conditions. Formal assessments of gastrointestinal

symptoms or sleep conditions frequently associated with ASD were not conducted.

^**^**^One participant reported taking both an antidepressant and an anti-psychotic.

**Fig 1:**
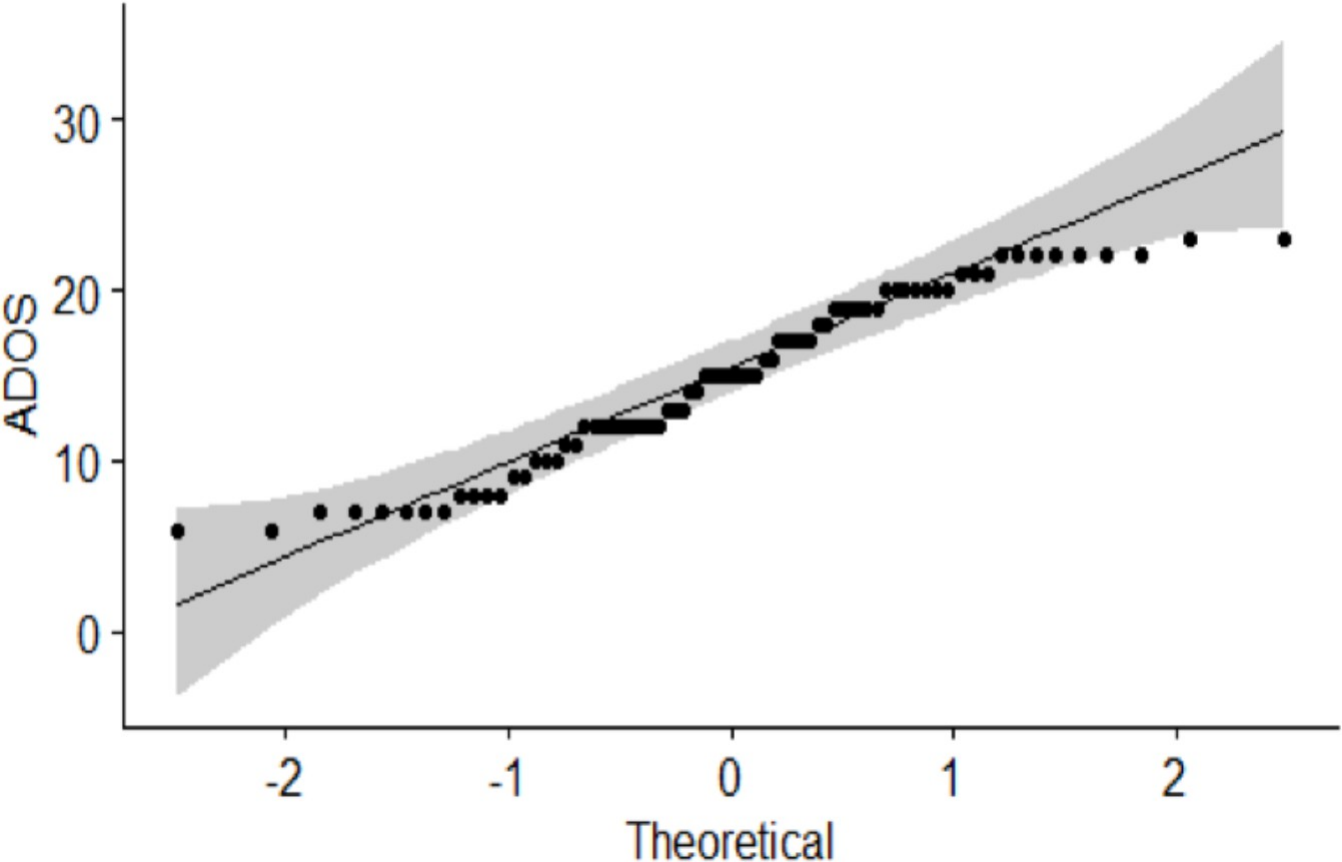
Range of ADOS scores among the ASD boys. Each dot represents the ADOS total score for each subject.

For the ASD group, all subjects were assessed by a clinical psychologist with research-reliability training using both the ADOS and the Autism Diagnostic Interview–Revised (ADI-R). A clinical diagnosis was made based on these data and overall clinical impression using DSM-5 criteria. In addition, ADOS diagnostic algorithms consisting of two behavioral domains: Social Affect (SA) and Restricted and Repetitive Behaviors (RRB) were used to determine an ADOS total score, which provides a continuous measure of overall ASD symptom severity. These scores can be used to compare ASD symptom severity across individuals of different developmental levels [29, 30] and were used in the correlation analyses (Fig 1).

For the TD group, all subjects underwent a developmental screening using the Adaptive Behavior Assessment System ‐ Second Edition (ABAS-II) to rule out developmental concerns. TD subjects were excluded if they had any first- or second-degree relatives diagnosed with ASD.

### Blood collection and storage

All subjects were healthy, defined as being fever-free for 24 hours, and presenting with no clinical symptoms. A fasting blood draw was performed on ASD and TD subjects between the hours of 8–10 am in a 3.5 ml Serum Separation Tube using standard venipuncture technique. The blood was gently mixed by 5 inversions and then stored upright for clotting at room temperature for 10–15 min. Blood was centrifuged immediately after the clotting time in a swing bucket rotor for 15 min at 1,100–1,300 g. After centrifugation was completed and the turbidity and hemolysis of the serum had been recorded, 250 μl aliquots of serum were transferred to 1.0ml coded cryovials and then stored at -80°C. Serum was shipped on dry ice to SomaLogic (Boulder, CO) for analysis.

### SOMAScan^TM^

The SOMAScan^TM^ platform 1.3k was used for analysis, and assays were run by SomaLogic. SOMAmer aptamer reagents consist of short single-stranded DNA sequences with ‘protein-like’ appendages that allow tight and specific binding to protein targets.

### Bioinformatics

The assay measured 1,317 proteins in 150 μl serum in 154 samples to identify an optimal subset of proteins to be used as a panel for ASD prediction. An additional 14 samples (7 ASD and 7 TD) were included as blinded duplicates to assess the variability of SOMAScan^TM^ analytes. In this study, 192 proteins failed to pass quality control (QC). After removing these proteins, 1,125 proteins were analyzed. The protein abundance data were normalized by taking log10 transform and then z-transformation. To deal with outliers, any z-transformed values less than -3 and greater than 3 were clipped to -3 and 3, respectively. To discover proteins for ASD prediction, three different methods were deployed: random forest (RF), t-test, and correlation-based methods.

- RF, a well-known decision tree-based ensemble learning method, produces consistent results even without hyper-parameter tuning. At the same time, it measures feature importance by observing how random re-shuffling of each predictor influences its model performance. To train RF models and calculate feature importance, an R package, ‘Random Forest’, was used. In this study, we chose MeanDecreaseGini (mean decrease in Gini Index), a weighted measure of the average reduction in node impurity within a RF, as the surrogate representing a protein’s importance in predicting ASD versus TD. With the normalized data, we trained an RF model 100 times. Each protein’s importance value was then averaged over the 100 runs. The 10 proteins with the highest averaged importance values were chosen for the RF-based prediction model.
- A t-test, which determines if there is a significant difference between the means of two groups, is a widely used approach to discover biomarkers in biological data. In this study, the 10 proteins with the most highly significant t-test values were selected for the prediction model.
- A correlation approach, which measures the statistical relationship between two variables, was used to calculate each protein’s correlation with ADOS total scores (SA + RRB), as a measure of ASD severity. Based upon the absolute values of each protein’s correlation coefficient, the 10 most highly correlated proteins were selected as the correlation-based predictive proteins.

After identifying the top-10 predictive proteins from each of the 3 methods (RF, t-tests and correlation), we found 6 proteins common to RF and t-test methods (highlighted). These 6 proteins were considered ‘core’ proteins, leaving 18 additional proteins that were not part of the core. None of the top-10 predictive proteins for the correlation method overlapped with those identified with the RF and t-test methods. A prediction model trained with the 6 core proteins was taken as a baseline model. Next, we investigated whether the addition of one or more of the remaining 18 proteins provided any additive predictive power.

A logistic regression model was used with datasets based upon the RF model, the t-test model and the correlation model, taking the subjects’ assigned group (ASD or TD) as output variables. We randomly assigned 80% of subjects to a training dataset and the remaining 20% of subjects to a test dataset. We then calculated the trained model’s area under the curve (AUC) for the test dataset as an evaluation metric. This process was repeated 1,000 times so as to obtain a rigorous evaluation while suppressing any bias which might be caused by favorable data splits.

A pathway enrichment analysis was performed for the optimal proteins. Entrez Gene IDs corresponding to the optimal proteins were fed to a *limma*::*goana* function in R. From its gene ontology results, the Top-20 biological process pathways were extracted and reported.

Finally, to evaluate possible confounding factors, we examined the impact of ethnicity, co-morbid conditions/clinical diagnoses, age, and medication use (Table 1) on the 12 proteins. To test the effect of ethnicity the data were split into two groups, white (n = 73) and non-white (n = 77) subjects. Four participants did not provide any information regarding ethnicity.To test the effect of allergies, the only clinical diagnosis with sufficient numbers for testing, the data were split into two groups, participants with allergies (n = 44) and those without (n = 105). Five participants did not provide any information regarding co-morbid conditions. T-tests were then run to compare the two modified datasets for each of the core proteins. To test the effect of age, a Spearman’s rank correlation was run for each protein against the age distribution of subjects. To test the effect of psychiatric medications, the AUC values for the full data set (n = 154) were compared with the AUC values of the dataset without the 8 subjects reporting medication usage (n = 144). Two participants did not provide any information regarding medication use.

## Results

**Fig 2:**
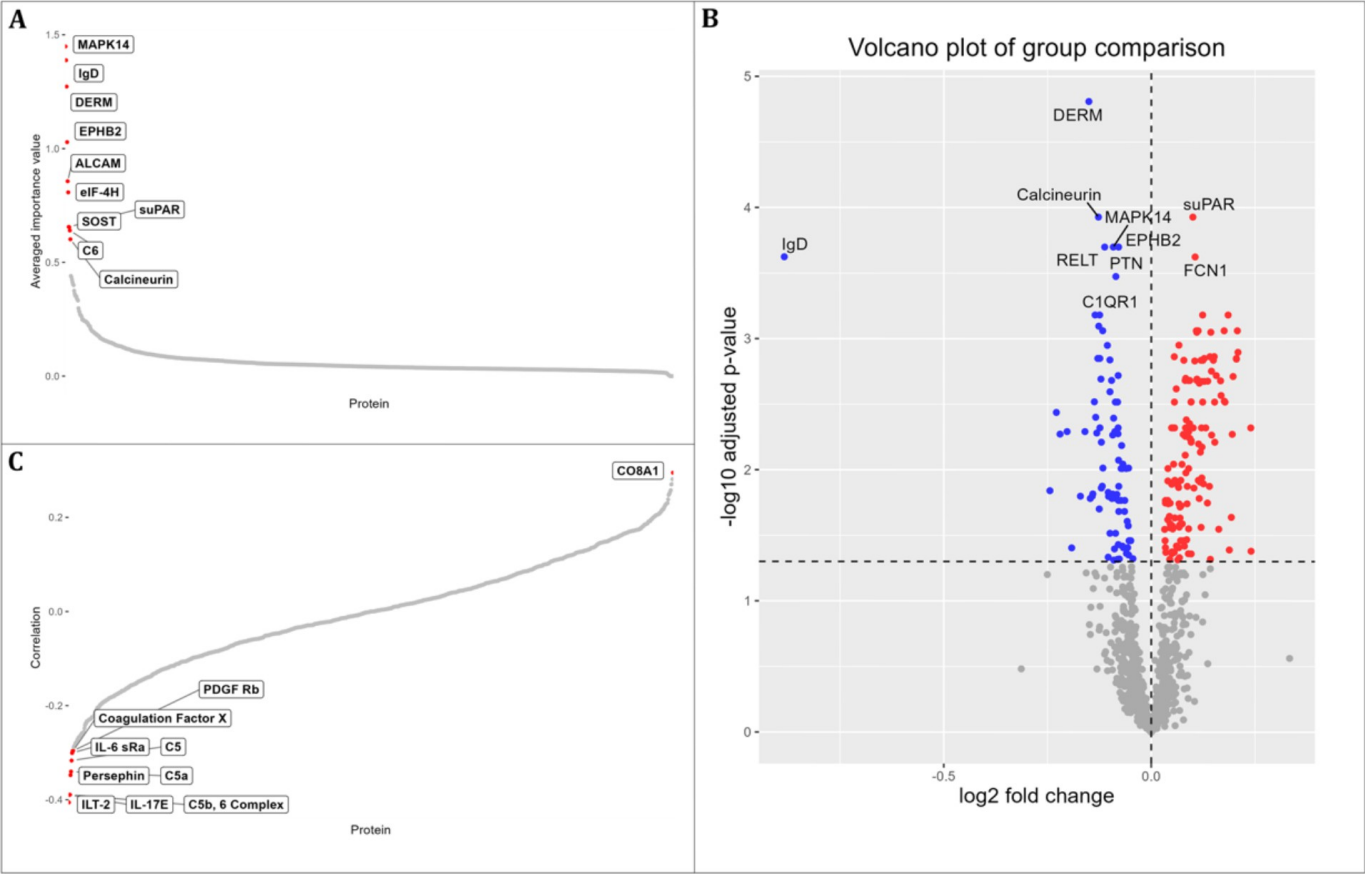
Top-10 predictive proteins identified by three different methods. (A) Random Forest-based method. (B) T-test based method. A total of 86 proteins were downregulated in the ASD boys and 52 proteins were upregulated. All of these proteins had a false discovery rate <0.05. (C) Correlation-based method.

A total of 1,125 proteins identified using the SomaLogic SOMAScan^TM^ platform were included in the analyses. Three computational methods were combined to search for a panel of proteins with high predictive power for ASD. The top-10 proteins were sought using RF analysis, t-test analysis between ASD and TD groups, and a correlation analysis with ASD severity (Fig 2). Six proteins were shared between RF and t-test prediction models used: mitogen-activated protein kinase 14 (MAPK14), immunoglobulin D (IgD), dermatopontin (DERM), ephrin type-B receptor 2 (EPHB2), soluble urokinase-type plasminogen activator receptor (suPAR), and calcineurin. These 6 proteins were defined as core proteins (Table 2).

**Table 2:** Top-10 predictive proteins identified by three different methods. The 6 core proteins common to Random Forest and t-test methods are highlighted.

No.	Random Forest		T-test			Correlation with ADOS total scores		
	Protein	Importance value	Protein	log2 fold change	p-value	Protein	Coefficient	p-value
1	MAPK14	1.4489	DERM	-0.1505	1.3837e-08	ILT-2	-0.4058	2.7609e-04
2	IgD	1.3883	suPAR	0.1000	2.5238e-07	IL-17E	-0.3898	5.0051e-04
3	DERM	1.2726	Calcineurin	-0.1274	3.1577e-07	C5b, 6 Complex	-0.3895	5.0651e-04
4	EPHB2	1.0284	MAPK14	-0.0916	1.0691e-06	Persephin	-0.3477	2.0839e-03
5	ALCAM	0.8565	EPHB2	-0.0788	8.8167e-07	C5a	-0.3402	2.6359e-03
6	eIF-4H	0.8077	RELT	-0.1123	1.0065e-06	C5	-0.3168	5.2971e-03
7	suPAR	0.6558	FCN1	0.1056	1.6464e-06	IL-6 sRa	-0.3008	8.2900e-03
8	SOST	0.6543	IgD	-0.8843	1.6952e-06	PDGF Rb	-0.2966	9.2736e-03
9	C6	0.6403	PTN	-0.0855	2.6899e-06	Coagulation Factor X	-0.2958	9.4731e-03
10	Calcineurin	0.6015	C1QR1	-0.1245	7.6353e-06	CO8A1	0.2950	9.6870e-03

**Fig 3:**
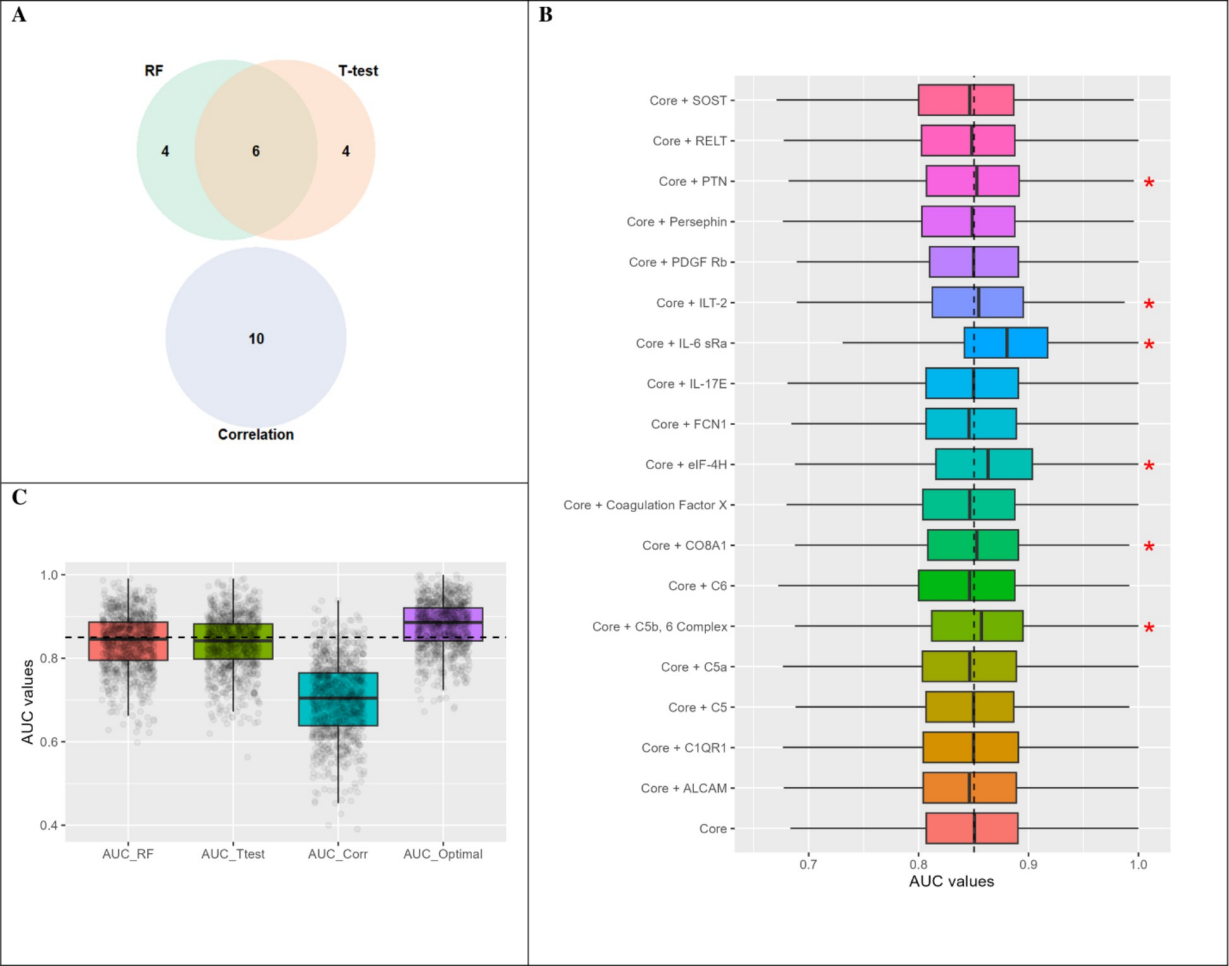
Optimization of the predictive proteins. (A) Identification of the 6 core proteins. The core proteins were among the top-10 proteins identified by RF and t-test methods. (B) Identification of 6 proteins with additive predictive power when combined with the core proteins (red asterisks). (C) Predictive power (AUC) of the top-10 proteins from the three different methods: RF–random forest, Ttest–t-tests, and Corr–correlation compared with the optimal panel of 12 proteins (AUC_Optimal) representing the 6 core proteins and the 6 additional proteins identified in Fig 3B).

In order to optimize the predictive power of the biomarker panel we first sought whether there was any protein overlap among the three methods (Fig 3A). There were 6 core proteins that were common to RF and T-test methods. Each of the additional Top-10 predictive proteins (n = 18) was successively added to the core proteins, one at a time, to see if they increased the predictive value of the AUC using logistic regression (Fig 3B). Six additional proteins: Pleiotrophin [PTN], Immunoglobulin-like transcript 2 [ILT-2], Interleukin-6 receptor subunit, [IL-6 rSa], eukaryotic translation initiation factor 4H [elF-4H], Collagen Type VIII Alpha 1 Chain [CO8A1] and Complement C5b, 6 Complex [C5b, 6 Complex], increased the AUC when each was added to the core proteins (Fig 3B). The AUC for the top-10 proteins identified by each model was: RF = 0.839±0.066, t-test = 0.837±0.066 and correlation = 0.701±0.089. Combining the 6 core proteins with the additional 6 proteins resulted in an AUC = 0.8790±0.0572, with a sensitivity = 0.8324±0.1137, and specificity = 0.8530±0.1076 (Fig 3C), and represents the 12 optimal proteins (AUC_Optimal).

The top-20 biological processes from pathway enrichment analysis are shown in Table 3. The 12 optimal proteins have pathway significance related to a number of processes associated with immune function in ASD, for example.

**Table 3:** Top-20 GO Terms from pathway enrichment analysis.

GO Term	GO Term	Total genes	Genes in list	Related optimal proteins	p-value
GO:0002682	regulation of immune system process	1488	9	C5b, 6 Complex; MAPK14; EPHB2; IgD; IL-6 sRa; Calcineurin; PTN; ILT-2	7.20E-08
GO:0002684	positive regulation of immune system process	1029	8	C5b, 6 Complex; EPHB2; IgD; IL-6 sRa; Calcineurin; PTN; ILT-2	8.34E-08
GO:0001819	positive regulation of cytokine production	489	6	C5b, 6 Complex; MAPK14; EPHB2; IgD; IL-6 sRa; ILT-2	4.31E-07
GO:0002460	adaptive immune response based on somatic recombination	311	5	C5b, 6 Complex; EPHB2; IL-6 sRa; ILT-2	1.33E-06
GO:0006935	chemotaxis	614	6	C5b, 6 Complex; MAPK14; EPHB2; IL-6 sRa; suPAR; PTN	1.63E-06
GO:0042330	taxis	616	6	C5b, 6 Complex; MAPK14; EPHB2; IL-6 sRa; suPAR; PTN	1.66E-06
GO:0043271	negative regulation of monoatomic ion transport	140	4	EPHB2; Calcineurin; ILT-2	1.90E-06
GO:0032101	regulation of response to external stimulus	1080	7	C5b, 6 Complex; MAPK14; EPHB2; IL-6 sRa; suPAR; PTN; ILT-2	2.56E-06
GO:0048584	positive regulation of response to stimulus	2290	9	C5b, 6 Complex; IgD; IL-6 sRa; suPAR; Calcineurin; PTN; ILT-2	2.91E-06
GO:0002250	adaptive immune response	693	6	C5b, 6 Complex; EPHB2; IgD; IL-6 sRa; ILT-2	3.29E-06
GO:0051240	positive regulation of multicellular organismal process	1667	8	C5b, 6 Complex; MAPK14; EPHB2; IgD; IL-6 sRa; Calcineurin; PTN; ILT-2	3.36E-06
GO:0048583	regulation of response to stimulus	4097	11	C5b, 6 Complex; MAPK14; EPHB2; IgD; IL-6 sRa; suPAR; Calcineurin; PTN; ILT-2	3.61E-06
GO:0010628	positive regulation of gene expression	1192	7	C5b, 6 Complex; MAPK14; EPHB2; IgD; IL-6 sRa; Calcineurin; ILT-2	4.95E-06
GO:0001568	blood vessel development	782	6	C5b, 6 Complex; CO8A1; MAPK14; EPHB2; IL-6 sRa; Calcineurin	6.60E-06
GO:0030155	regulation of cell adhesion	803	6	CO8A1; MAPK14; EPHB2; suPAR; Calcineurin; ILT-2	7.69E-06
GO:0001944	vasculature development	815	6	C5b, 6 Complex; CO8A1; MAPK14; EPHB2; IL-6 sRa; Calcineurin	8.37E-06
GO:0001817	regulation of cytokine production	847	6	C5b, 6 Complex; MAPK14; EPHB2; IgD; IL-6 sRa; ILT-2	1.04E-05
GO:0001816	cytokine production	860	6	C5b, 6 Complex; MAPK14; EPHB2; IgD; IL-6 sRa; ILT-2	1.14E-05
GO:0050776	regulation of immune response	863	6	C5b, 6 Complex; MAPK14; EPHB2; IgD; ILT-2	1.16E-05
GO:0002376	immune system process	2702	9	C5b, 6 Complex; MAPK14; EPHB2; IgD; IL-6 sRa; Calcineurin; PTN; ILT-2	1.17E-05

To determine the accuracy of the SomaScan^TM^ assay we ran duplicate blood samples from 14 subjects (7 ASD and 7 TD). The 12 proteins selected for the optimal ASD biomarker panel exhibited an average of 6 to 13% variability between the duplicate assays.

Finally, ethnicity, allergies, age, and medication use were analyzed as independent variables using t-tests or Spearman’s rank correlation, as appropriate (S1 Table). Ethnicity was significantly associated with IL-6 sRA (p = 0.02517006) and ILT-2 (p = 0.01750944) protein counts, whereas participants reporting allergies showed significantly lower IgD (p = 0.01235089) protein counts compared to those reporting no allergy. For age, all of the correlation coefficients were small (r = -0.22 to 0.35; S1 Table), indicating there is no age effect on protein counts. The use of psychiatric medication did not significantly impact the AUC for the optimal proteins: the AUC for the total dataset was 0.8790 ±0.0572, whereas the AUC for the dataset with the 8 subjects reporting medication use removed was 0.8654±0.0626.

## Discussion

The goal of the present study was to identify a blood biomarker profile for ASD from >1,200 proteins using the SOMAScan^TM^ platform. Twelve proteins were identified based upon a novel combination of machine learning methods with random forest analysis, t-test analysis, and correlation analysis with ADOS total scores that produced an accurate identification of ASD in boys. Six of the proteins, IgD, suPAR, MAPK14, EPHB2, DERM, and calcinerin were present in RF and t-test analyses and were considered core proteins in the panel. Another 6 proteins providing additive power were combined with the 6 core proteins, and together, the 12 proteins resulted in an AUC of 87% (sensitivity 83%; specificity 85%). These proteins have pathway significance related to a number of processes, including regulation of immune system process, positive regulation of cytokine production, adaptive immune response, regulation of cytokine production, and cytokine production, which have previously been associated with ASD [31]. Age and use of psychiatric medication did not impact the protein counts for the biomarker panel. Ethnicity was significantly associated with IL-6 sRA and ILT-2 protein counts.

Immune system aberrations have been reported in ASD for some time [32]. Abnormalities in serum antibody concentrations and T cell responses have been well described [33–35]. Altered cytokine profiles [36–38], decreased immunoglobulin levels, particularly IgG [39], altered cellular immunity [40], and neuroinflammation [41] in ASD have been consistently identified. Furthermore, autoimmunity has been implicated in ASD, with several studies reporting circulating autoantibodies to neural antigens [42, 43]. More recently, ASD biomarker studies identified significant dysregulation of genes involved in immune function and inflammation [44, 45]. Out of the 6 core proteins in the panel, IgD exhibited the greatest difference between ASD and TD samples. IgD was 58% lower in ASD serum compared with TD serum. While there is little information on the role of IgD in ASD, increased levels have been reported in a mouse model of systemic lupus erythematosus [46], an autoimmune disease, thus IgD may have a role in inflammation. Another core protein, soluble urokinase plasminogen activator receptor (suPAR), was found to be 16.4% higher in ASD serum compared with TD serum. suPAR, the soluble form of uPAR, which is expressed on neutrophils, activated T-cells, and macrophages [47], is released during inflammation or immune activation. suPAR is a biomarker of inflammation in critically ill patients, although elevated levels of suPAR have also been reported over a wide range of clinical conditions [48]. suPAR is thought to be involved in the modulation of cell adhesion, migration, and proliferation pathways [49]. It, therefore, follows that elevated suPAR may affect cell adhesion processes, neuronal migration, and proliferation in the developing brain contributing to ASD [50, 51]. While further studies are needed to understand the role of suPAR in the etiology of ASD, children who reported ‘adverse childhood experiences’ had lower IQ scores or poorer self-control and showed elevated suPAR levels as adults [52].

A third core protein, mitogen-activated protein kinase 14 (MAPK14), was significantly lower in ASD versus TD serum. MAPK14 is activated in response to stress and inflammation [53]. In two studies examining gene expression profiles in blood samples from children with and without ASD, MAPK14 was differentially expressed–one of only 5 genes that overlapped between the two studies [45, 54]. The MAPK pathway is important in neural development, learning, and memory in syndromic conditions associated with ASD, such as tuberous sclerosis and Smith-Lemli-Opitz disorder [55]. Although the roles of IgD, suPAR, and MAPK14 in ASD are not well understood, alterations in immune response and/or inflammatory pathways have been implicated in many studies of children with ASD and remain a target of interest for many biomarker studies.

Another core protein, EPHB2, is linked to NMDA glutamate receptor activity. Interestingly, several lines of evidence suggest an imbalance between excitatory (glutamate-mediated) and inhibitory (GABA-mediated) neurotransmission, which may be a common pathophysiological mechanism and treatment target for ASD [56–59]. Finally, another core protein, Calcineurin, a phosphatase important for synaptic plasticity and neuronal development, has been implicated in the etiology and pathophysiology of neuropsychiatric disorders, including intellectual disability, ASD, epilepsy, and Alzheimer’s disease [60, 61].

While none of the top-10 proteins from the correlation analysis overlapped with those from the RF or T-test analyses, four of the six additive proteins (ILT-2, IL-6 sRa, CO8A1, and C5b, 6 Complex) were top-10 proteins from the correlation analysis indicating that some of the biomarker proteins in the panel were associated with ASD severity, as measured by ADOS total scores.

In a previous study, we investigated 110 proteins using the MesoScale Discovery platform, and two proteins were found to be most important: IL-8 and TSH [10]. These proteins have been identified as putative ASD biomarkers in other studies [62–64]. Similar to our previous report, in the current study, IL-8 levels were significantly higher (23%, p = 0.002) and TSH levels were significantly lower (67%, p = 0.007), when comparing ASD to TD boys (S2 Table). Because the present study searched >1,200 proteins to find those most important for identifying ASD, IL-8 and TSH, though significantly different between ASD and TD, were not among those with the highest t-test values or importance as measured by RF.

There are some limitations to the present study. Although the sample size is acceptable for a discovery study, the data presented here are preliminary, and a larger validation study is needed to be certain of the value of the biomarker panel. Due to the increased prevalence of ASD in boys, this study only enrolled boys, which does not allow for an investigation of gender-specific differences. Furthermore, although there was no effect of age on the panel of proteins selected, a prospective study is planned that would shed light on the stability of these proteins over time. When making aptamer measurements on SomaScan^TM^ plates containing >1,200 protein markers/well, well-to-well differences may add variability to the data. To address this, we ran duplicate samples on a subset of ASD and TD samples to determine the variability of the measurements, and for the 12 optimal proteins, the variability in measurement for each protein was <14%. The complex phenotypic heterogeneity of ASD also presents some limitations when performing biomarker studies. To address this, we used standardized diagnostic criteria to classify individuals with ASD, as well as incorporating analyses of ethnicity, co-morbid conditions/clinical diagnoses, and medication use. The ADOS can only be used to assess behaviors in children with ASD so the correlation analyses did not include controls. As such, none of the top-10 proteins identified by the correlation analysis overlapped with those from the RF and t-test analyses. Co-morbid conditions, such as anxiety, epilepsy, ADHD, and gastrointestinal disorders, occurred at very low frequencies in our cohorts and could not be analyzed. The most commonly reported clinical diagnosis, allergies, was significantly correlated with IgD protein counts. Medication use had no effect on the core proteins. Finally, dietary intervention and use of nutritional supplements were difficult to assess accurately due to the limited information collected and our inability to verify the data provided, especially since some subjects were not under the care of a nutritionist or dietitian at the time of study participation.

## Conclusions

The present study used serum samples from ASD and TD boys to search for a panel of proteins with diagnostic accuracy for the identification of ASD. Over 1,100 proteins were examined on the SomaScan^TM^ platform. A panel of 12 proteins was identified using three computational methods: RF, t-testing, and correlation analysis with ASD severity scores. These 12 proteins were significantly different in ASD compared with TD boys, and several of these proteins have been mechanistically (suPAR, MAPK14, and EPHB2) and genetically (EPHB2 and suPAR) linked to ASD. The panel of proteins exhibited an AUC of 87% (specificity 85%; sensitivity 83%). This novel set of proteins has the potential to be an efficacious blood-based biomarker for the early identification of ASD in boys, particularly since behavioral and developmental assessments are not easily administered in very young children. While the use of machine learning for ASD diagnosis is still in its infancy, identifying key proteomic biomarkers may also lead to targeted intervention strategies as we further elucidate the functional processes associated with ASD and the mechanistic interplay between brain structure and behavior.

## Supporting information

S1 Table Analysis of the effect of ethnicity (t-test), allergies (t-test), and age (Spearman’s rank correlation) on optimal proteins. (DOCX)

S2 Table Comparison of IL-8 and TSH levels in ASD and TD boys. (DOCX)

S1 Dataset (XLSX)
