# Supplementary material for: Blood biomarker discovery for autism spectrum disorder: A proteomic analysis
Source: PLoS One. 2024 Dec 19;19(12):e0302951. doi: 10.1371/journal.pone.0302951 (PMC11658466; doi:10.1371/journal.pone.0302951)
Supplement: S1 Table — (DOCX) [file pone.0302951.s001.docx]

**S1 Table. Analysis of the effect of ethnicity (t-test), seasonal allergies (t-test), and age (Spearman Rank correlation) on the 12 optimal ASD biomarker proteins.**

|  | **Ethnicity** | **Allergies** | **Age** | |
| --- | --- | --- | --- | --- |
| **Optimal Protein** | **p-value** | **p-value** | **Correlation Coefficient** | **p-value** |
| C5b, 6 Complex | 0.64072974 | 0.72797713 | 0.04884572 | 0.54745365 |
| Calcineurin | 0.05125406 | 0.30348319 | -0.16620172 | 0.03939324 |
| CO8A1 | 0.73716232 | 0.35612015 | 0.06996585 | 0.38855727 |
| DERM | 0.0996302 | 0.91552217 | 0.13890995 | 0.08577430 |
| eIF-4H | 0.40664300 | 0.35337948 | -0.08257859 | 0.30859990 |
| EPHB2 | 0.88663763 | 0.39141576 | -0.22595306 | 0.00483700 |
| IgD | 0.34035058 | 0.01235089 | 0.35625937 | 0.00000577 |
| IL-6 sRa | 0.02517006 | 0.98818108 | 0.02502088 | 0.75806752 |
| ILT-2 | 0.01750944 | 0.82724494 | -0.10103523 | 0.21247376 |
| MAPK14 | 0.90311703 | 0.29072532 | -0.00884615 | 0.91329322 |
| PTN | 0.98858169 | 0.43954503 | -0.14668443 | 0.06947672 |
| suPAR | 0.07456119 | 0.62255598 | -0.09075171 | 0.26299723 |
