# Supplementary material for: Blood biomarker discovery for autism spectrum disorder: A proteomic analysis
Source: PLoS One. 2024 Dec 19;19(12):e0302951. doi: 10.1371/journal.pone.0302951 (PMC11658466; doi:10.1371/journal.pone.0302951)
Supplement: S2 Table — (DOCX) [file pone.0302951.s002.docx]

**S2 Table. Comparison of mean IL-8 and TSH levels (normalized) in ASD and TD boys.**

|  | | **T-test** | |
| --- | --- | --- | --- |
| **Group** | **Protein** | **Mean (normalized)** | **p-value** |
| ASD | IL-8 | -0.1763751 | 0.002 |
| TD | IL-8 | -0.2291225 |  |
| ASD | TSH | 0.06342079 | 0.007 |
| TD | TSH | 0.1956321 |  |
